# Feeding amount significantly alters overt tumor onset rate in a zebrafish melanoma model

**DOI:** 10.1242/bio.030726

**Published:** 2018-01-15

**Authors:** Vadim Grigura, Megan Barbier, Anna P. Zarov, Charles K. Kaufman

**Affiliations:** 1 Division of Medical Oncology, Department of Medicine and Department of Developmental Biology, Washington University in Saint Louis, 4518 McKinley Ave., CB 8069, St. Louis, MO 63110, USA; 2 School of Arts and Sciences, Washington University in Saint Louis, 1 Brookings Drive, St. Louis, MO, 63130, USA

**Keywords:** Melanoma, Cancer models, Metabolism, Obesity, Caloric restriction

## Abstract

The manner in which zebrafish are fed may have important impacts on the behavior of disease models. We examined the effect of different feeding regimens on the rate of overt melanoma tumor onset in a *p53/BRAF*-dependent model, a commonly used read-out in this and many other cancer models. We demonstrate that increased feeding leads to more rapid melanoma onset. The ability to modulate overt tumor onset rates with this regimen indicates additional flexibility to ‘tune’ the system to more quickly generate tumors for study and to begin to address questions related to cancer metabolism using the zebrafish model.

## Abstract (precis)

**Summary:** The rate of tumor formation in a BRAF-driven zebrafish melanoma model can be significantly altered by increased feeding and opens a new avenue for studying the underlying metabolic pathways involved.

## INTRODUCTION

Genetic models of cancer are dependent on defined driver oncogenes and tumor suppressors, thus creating a homogeneous population in which to study the effect of chosen perturbations on cancer formation. One limitation of such genetically engineered adult tumor models is that there may be a delay to onset of detectable tumors, which slows analysis, adds costs for housing, and can fill available facility space. The long-recognized association between food/caloric intake and longevity/overall health, including rates of cancer, in many organisms (Ravussin et al., 2015; Mattison et al., 2017; Colman et al., 2014; Klass, 1977; Bross et al., 2005; Weindruch and Walford, 1982) led us to wonder if altered feeding of a favored zebrafish melanoma model (i.e. *p53/BRAF* model) would alter the rate of grossly detectable tumor onset (Patton et al., 2005; Ceol et al., 2011; White et al., 2011; Lian et al., 2012; Kaufman et al., 2016).

## RESULTS AND DISCUSSION

**Fig. 1.**
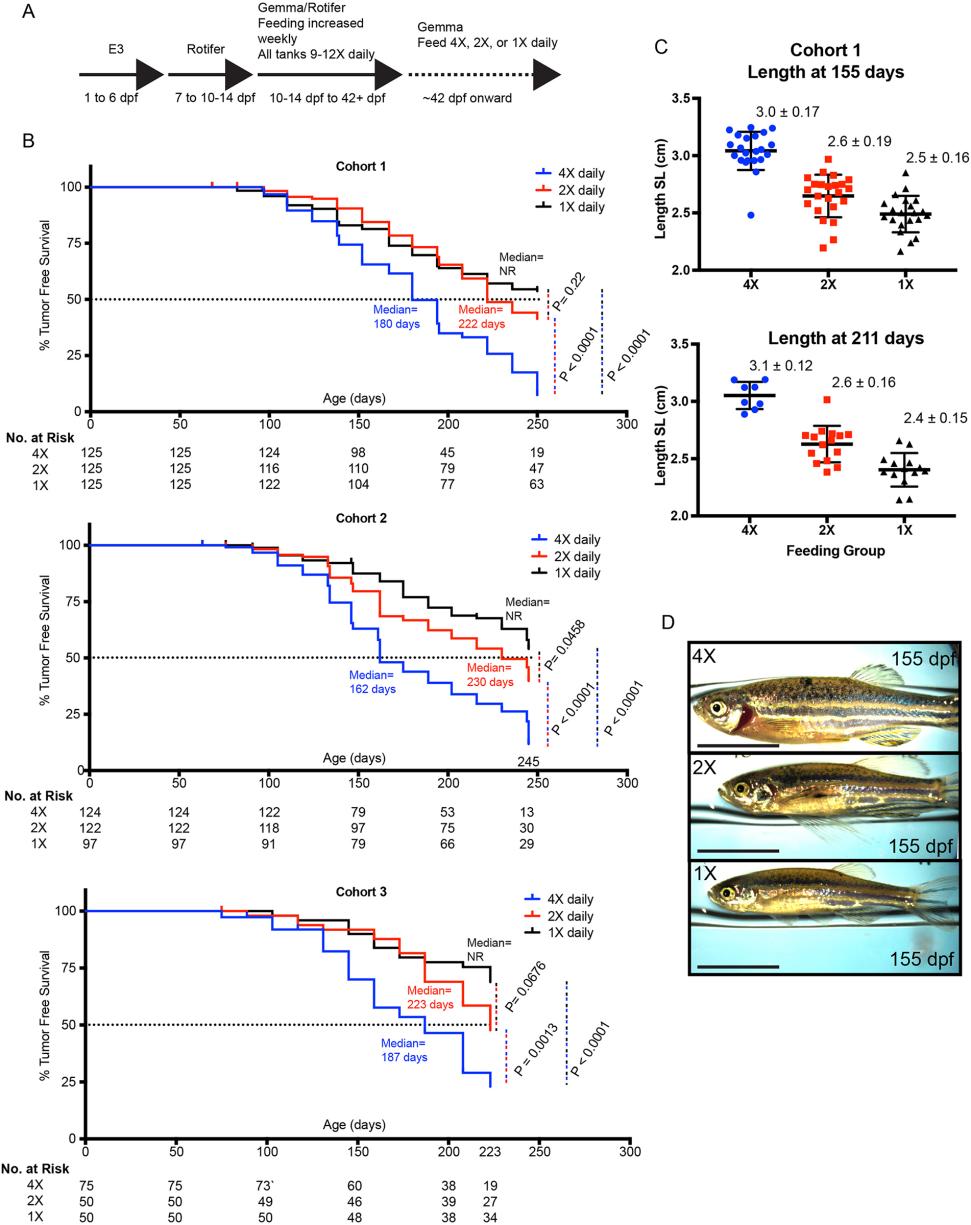
**Feeding amount alters melanoma tumor onset in zebrafish *p53/BRAF* model.** (A) Feeding paradigm used to rapidly rear *p53/BRAF* melanoma-prone zebrafish. (B) Gross tumor onset reported as Kaplan–Meier curves for percent (%) tumor-free survival at the indicated age (dpf). Number of zebrafish at risk are reported at 50 day intervals based on the most proximal prior observation. Reported *P* values are based on the Log-rank (Mantel-Cox) Test as determined by Prism software where curves were generated. NR, not reached. (C) Zebrafish length at the indicated times for various adult feeding regimens. SL, snout to caudal peduncle length, is reported in centimeters with mean±standard deviation. (D) Representative images of *p53/BRAF* zebrafish at 155 dpf in each feeding group. Scale bar: 1 cm.

Our zebrafish facility employs an aggressive feeding protocol using Tecniplast Tritone robotic feeders throughout the fish life-span (Fig. 1A), allowing for rapid rearing of zebrafish as our standard protocol. The use of the Tritone feeders also allows for precise and simple manipulation of daily feeding programs. Parental fish homozygous for the melanoma-inducing *p53/BRAF* mutations were incrossed, and 50 embryos were placed per 10 cm petri dish in E3 buffer until 6 days postfertilization (dpf) (Fig. 1A). Some parental fish carried the *Tg(crestin:EGFP)* transgene, but this was not analyzed for this study (Kaufman et al., 2016). Each dish was then reared in a 3.5 liter tank with excess rotifers beginning at 6 dpf until 10-14 days of life. Developing juvenile zebrafish were then fed via Tritone robot increasing aliquots of GEMMA pellet (Skretting)±rotifer culture (Fig. 1A; Table S1). Importantly, to minimize any ‘jackpot’ effects of single tanks, all juvenile zebrafish born on the same day were mixed during 4-5 weeks of life, and then randomly redistributed with 25 individuals per 3.5 liter tank. These tanks were then moved from the nursery to the main system during the sixth week of life and assigned a feeding label providing four (4X), two (2X), and one (1X) time daily feeding of the same weight (60 mg) of GEMMA pellet per feeding (Fig. 1A).

As previously described, visual inspection of *p53/BRAF* zebrafish for raised lesions identifies melanoma tumors reproducibly, and this approach has been used to establish the role of multiple factors in modifying melanomagenesis (Ceol et al., 2011; Lian et al., 2012). Thus, each cohort of zebrafish was scored for melanoma onset every 2 weeks beginning at 9-10 weeks of age. The total number of animals in each tank was kept constant by replacing any tumor-bearing zebrafish, which were removed upon identification of a tumor, with similarly aged *Casper* or *Na* zebrafish. As shown in Fig. 1B, the rate of detectable melanoma was significantly increased in three independent cohorts of *p53/BRAF* melanoma-prone zebrafish fed four times daily (4X, median onset 180, 162 and 187 days) as compared to those fed two (median onset 222, 230 and 223 days) or one time daily (median not reached in any cohort). These were highly significant differences comparing the four versus two times daily (*P*<0.0001, *P*<0.0001 and *P*<0.0013) and four versus one time daily (*P*<0.0001 in each cohort) schedules. There was also a trend of quicker onset in the two versus one daily groups, although not significant in all cohorts (*P*=0.22, *P*=0.0458 and *P*=0.0676). As all cohorts were fed our standard diet in the nursery, we did not intensively investigate effects on sexual maturity. The appearance of the melanomas in all groups at the time of tumor identification was qualitatively similar, and in this study, we did not track the behavior of established tumors in the different feeding groups. We measured a snapshot of size [(i.e. snout to tail, SL, length (Parichy et al., 2009)] of zebrafish in Cohort 1 at 155 and 211 days of life and found stable differences in size between the 4X and 2X/1X daily feedings (Fig. 1C,D).

Overall, our data reveal a significant modulation of gross melanoma tumor onset by the amount of feeding of adult zebrafish. Published datasets fit the consensus that the *p53/BRAF* melanoma model [e.g. median onset of ∼35 weeks (Neiswender et al., 2017) and not reached at 18 months (Lister et al., 2014)] typically produces tumors more slowly than the *p53/BRAF/Na/MiniCoopR* model [e.g. median onset of ∼19 weeks for controls (Lian et al., 2012; Ceol et al., 2011)]. In this study, aggressive rearing in the nursery and 4X daily feeding of adults achieved median onset of ∼25 weeks, approaching the speed of the *MiniCoopR* system (Fig. 1B). In humans and in mouse models, there does appear to be a link between obesity and melanoma formation and aggressiveness, consistent with potential modulation of melanoma behavior by obesity-linked metabolic changes (Clement et al., 2017). A number of studies have also found an important role for diet composition (i.e. high-fat content, antioxidants) and caloric intake on melanoma metastasis and growth, but in the setting of murine transplantation models (Piskounova et al., 2015; Erickson et al., 1979; Erickson, 1984; Rofe et al., 1985; Ershler et al., 1986; Malvi et al., 2014; Xia et al., 2017). Perhaps most intriguingly in this study, our current feeding paradigm supports the possibility of applying the molecular genetic toolbox offered by the zebrafish to rapid mechanistic studies of the relationship of diet, caloric intake, and metabolism to *de novo* melanoma tumor formation.

## MATERIALS AND METHODS

### Zebrafish husbandry

Zebrafish of the *p53/BRAF/crestin:EGFP* genotype (Patton et al., 2005; Kaufman et al., 2016) were maintained in a Tecniplast system with Tritone robotic feeding system at 28.5°C under standard housing protocols as per Institutional Animal Care and Use Committee (IACUC) regulations. GEMMA Micro 150 and 300 pellets were from Skretting, a Nutreco company. Details of the feeding protocol are provided in Fig. 1A and Table S1, and all experiments were performed under IACUC-approved protocols.

### Scoring of zebrafish for tumor formation

Every 2 weeks beginning at 9-10 weeks of age, zebrafish were briefly separated, five fish per holding tank, for counting, and then individually observed for presence of raised lesions, which are indicative of melanoma tumor formation as extensively described (Patton et al., 2005; Ceol et al., 2011; Kaufman et al., 2016). Those fish with melanoma tumors were removed and replaced with similarly age-matched *Casper* or *Nacre* fish, allowing for easy identification of ‘place-holder’ fish. Tumor onset data were entered into GraphPad Prism® to generate survival curves with *P* values based on the Log-rank (Mantel-Cox) Test.

## Supplementary Material

Supplementary information
