# Supplementary material for: Feeding amount significantly alters overt tumor onset rate in a zebrafish melanoma model
Source: Biol Open. 2018 Jan 15;7(1):bio030726. doi: 10.1242/bio.030726 (PMC5829507; doi:10.1242/bio.030726)
Supplement: Supplementary information [file biolopen-7-030726-s1.pdf]

Table S1 – Detailed Feeding Schedule for Nursery

Time	14-21 dpf	21-28 dpf	28-35 dpf	35-42 dpf+
**6:30am**	Dry	Dry	Dry	Dry
**7:45am**			Dry	Dry
**9:00am**	Dry	Dry	Dry	Dry
**10:30am**	Dry	Dry		Dry
**12:00pm**	Rotifers	Rotifers	Rotifers	Rotifers+Dry
**1:30pm**	Rotifers+Dry	Rotifers+Dry	Rotifers+Dry	Rotifers+Dry
**3:00pm**	Rotifers		Dry	Dry
**4:30pm**	Dry	Dry	Dry	Dry
**6:00pm**			Dry	Dry
**7:30pm**	Dry	Dry	Dry	Dry
**9:00pm**		Dry	Dry	Dry
**10:30pm**	Dry	Dry	Dry	Dry

Dry = 30 mg GEMMA Pellet (GEMMA Micro 150 for days 14-21, GEMMA Micro 300 after)
Rotifers = 11 ml rotifer culture

GEMMA Pellet composition per label: Fish meal, lecithin, wheat gluten, algae, fish oil, maize starch, vitamins, minerals

GEMMA Pellet Analyses per label: Protein 59%, Oils and Fats 14%, Fiber 0.2%, Ash 13%, Phosphorus 2.0%, Calcium 1.5%, Sodium 0.7%

GEMMA Pellet Additives per label: Vitamin A 23000 IU/kg, Vitamin D3 2800 IU/kg, Iron (ferrous sulphate monohydrous) 100 mg/kg, Iodine (calcium iodate anhydrous) 5.1 mg/kg, Copper (cupric sulfate pentahydrate) 10 mg/kg, Manganese (manganese sulfate monohydrate) 36 mg/kg, Zinc (zinc sulfate monohydrate) 130 mg/kg, Selenium (sodium selenite) 0.25 mg/kg, Selenomethionine produced by S. cerevisiae NCYC R397) 0.03 mg/kg

Biology Open • Supplementary information
